# Iatrogenic enterocutaneous fistula following an incarcerated Richter's femoral hernia misdiagnosed for an inguinal abscess: A case report

**DOI:** 10.1016/j.ijscr.2024.109736

**Published:** 2024-05-04

**Authors:** Baanitse Munihire Jeannot, Mugarura Anwar Biraali, Micheal Mugenyi, Richi Mukandirwa Wetemwami, Joshua Muhumuza, Franck Katembo Sikakulya

**Affiliations:** a Faculty of Clinical Medicine and Dentistry, Department of Surgery, Kampala International University Western Campus, Ishaka-Bushenyi, Uganda; b Faculty of Medicine, General Surgery Department, Université Catholique la Sapientia of Goma, Democratic Republic of the Congo; c Institut superieur de techniques medicales de Goma, Democratic Republic of the Congo

**Keywords:** Richter's femoral hernia, Iatrogenic EC fistula, Incarcerated hernia, Misdiagnosis, Case report

## Abstract

**Introduction and importance:**

As the Richter's hernia contains anti-mesenteric intestinal wall, patients usually do not present with obstructive symptoms. Consequently, this leads to delays in diagnosis and increased morbidity and mortality. Early detection and surgical treatment are therefore paramount to improving outcomes.

**Case presentation:**

A 51-year-old female presented with an incarcerated Richter's femoral hernia misdiagnosed as inguinal abscess that underwent incision and drainage. This developed into an enterocutaneous fistula (EC Fistula) and was eventually complicated by peritonitis, requiring laparotomy and herniorrhaphy. Post-operative recovery was uneventful.

**Clinical discussion:**

In advanced stages, Richter's femoral hernia may present with obstructive symptoms as in other incarcerated hernias. Richter's hernias may eventually present with obstructive symptoms in their advanced stages. Their relatively asymptomatic nature increases the risk of complications, such as enterocutaneous fistula.

**Conclusion:**

This case highlights how an incarcerated Richter's femoral hernia in a female misdiagnosed as an abscess delayed treatment, increased patient morbidity with development of an enterocutaneous fistula and peritonitis, and mandated surgical exploration to control sepsis and repair the hernia.

## Highlights

• The Richter’s hernia is a challenging condition that carries increased morbidity and mortality, especially when associated with an EC fistula.
• The characteristic lack of obstructive symptoms in a Richter’s hernia often delays diagnosis, leads to misdiagnosis, and ultimately worsens prognosis.
• The mainstay of treatment for a complicated Richter’s hernia entails surgical exploration, control of sepsis, and nutritional support.

## 1 Introduction

Richter's hernia is defined as “an abdominal hernia in which only part of the circumference of the bowel's anti-mesenteric border is entrapped in the hernial orifice” leading to ischemia, gangrene and perforation of the hollow viscus but without typical obstructive symptoms [1]. Only two thirds or less of the bowel wall circumference is involved, with often no features of complete intestinal obstruction. This leads to late diagnosis or even misdiagnosis [2]. Its asymptomatic nature increases the morbidity and mortality. Richter's hernia occurs at various positions with femoral ring being the most common [3].

Some patients with Richter's hernia may present with EC fistula either spontaneously or due to surgical intervention mistaking the obstructed hernia to be inguinal abscess [4]. The management of enterocutaneous fistulas remains complex and challenging.

Early surgery should be limited to abscess drainage or repair of associated hernias or dysfunctional proximal stomas [5].

We present a case of iatrogenic EC Fistula due to femoral Richter's hernia misdiagnosed as an inguinal abscess. The diagnosis procedure and management options in a resource limited setting are also discussed. The work has been reported in line with the SCARE criteria [6].

## 2 Case presentation

**Fig. 1:**
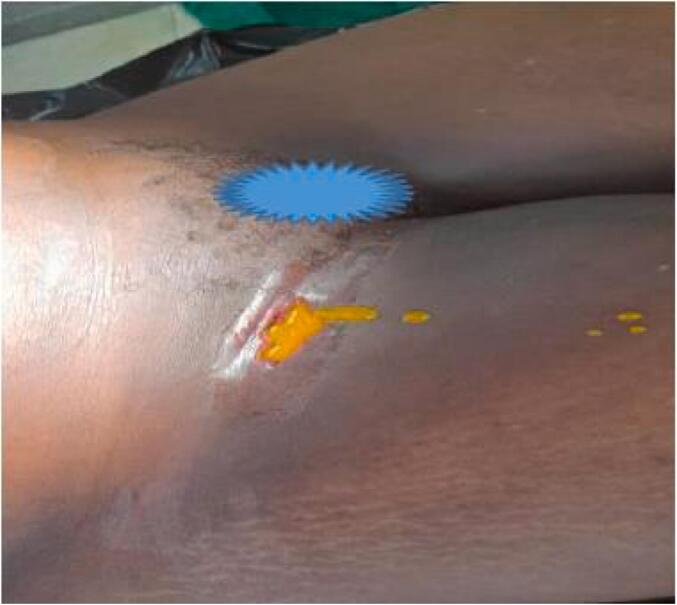
Showing yellowish discharge coming out from the right groin region.

A 51-year-old female with no significant medical problems and no prior surgeries presented to the emergency department complaining of an intermittent, yellowish discharge from the right groin for a period of two weeks (Fig. 1) associated with right sided, burning abdominal pain. She noted that she had right groin swelling for the past 3 months that was diagnosed as an inguinal abscess, which was therefore incised and drained. The wound did not heal and continued to intermittently drain yellow, feculent material.

Objectively, she was febrile to 38.6C with a blood pressure of 90/50 mmHg, pulse rate of 118 beats per minute, and oxygen saturation of 92 % on room air. She appeared acutely distressed and moderately dehydrated. Abdominal examination revealed tenderness to palpation, mild distension, dullness to percussion, and absent bowel sounds. A 3 cm incisional wound with expressible purulent discharge was noted in the right groin overlying a 4 cm diameter area of induration. There was no swelling palpated, and the discharge increased by increasing the abdominal pressure.

**Fig. 2:**
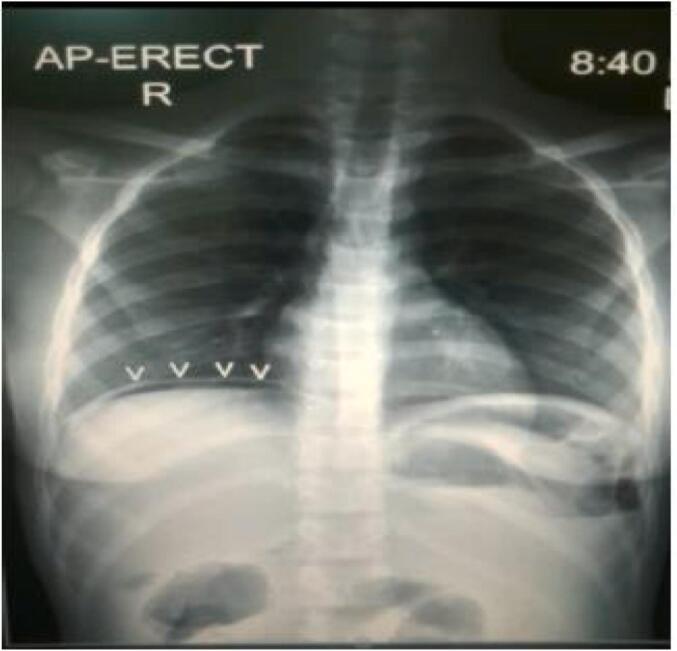
Revealed a pneumoperitoneum under the right hemidiaphragm.

**Fig. 3:**
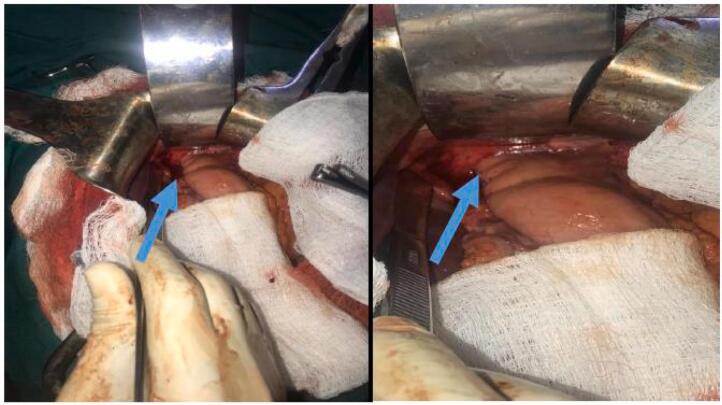
Ileum adhered to the femoral septum ring and entering the femoral canal in the hernia sac (blue arrow).

Rectal and pelvic examination was unremarkable. The patient was admitted for resuscitation and further workup. Labs were notable for leukocytosis and a hemoglobin of 6 g/dL, electrolytes, and liver function tests were within normal range. Abdominal ultrasound demonstrated that the inguinal wound was continuous with a loop of ileum incarcerated within the femoral space as well as peri-ileal fluid and a fluid collection in the hepatorenal space. Abdominal X-ray revealed pneumoperitoneum under the right hemidiaphragm (Fig. 2). The patient was diagnosed with peritonitis associated with an EC Fistula. An obstructed Richter's femoral hernia seemed likely to be misdiagnosed and treated as a groin abscess. A pre-operative blood transfusion was done in addition to Intravenous fluids. The patient was consented for exploratory laparotomy which was performed through a midline incision. On opening the abdomen, a distal ileum loop (about 25 cm proximal to ileocecal junction) was found to be incarcerated within the internal femoral ring, which was retrieved back into the abdominal cavity (Fig. 3). There was perforation and ischemia (around 6 cm) over the adhered loop. Resection of the necrotic segment of ileum was done with double barrel ileostomy.

The femoral hernia site was repaired by the classic repair approximating the inguinal ligament to Cooper's ligament and the pectineus fascia with Nylon suture. Mesh repair was not done for hernia defect due to faecal contamination. The internal femoral ring was additionally closed from inside of the peritoneal cavity with plicating sutures. The openings in the skin over the femoro-inguinal region were communicated with each other and has been left open due to cellulitis of the area involved and pus discharged. Six liters of normal saline were used to lavage the peritoneal cavity and a double burred ileostomy was performed, because of, bowel edema and peritonitis. After achieving adequate hemostasis and drain placement in pelvis, the abdomen was closed in layers. IV Antibiotics (ceftriaxone 2 g one time a D and metronidazole 500 mg three times a day for 7 days) and analgesics (tramadol 100 mg two times a day for 2 days then diclofenac tablet 50 mg two times a day for 3 days), IV fluids (3.5 L of Normal saline, ringer lactate and dextrose 5) and nutritional support was administered as planned. The open groin wound was debrided surgically and packed using Iodine before doing a delayed primary closure. We performed a delayed primary closure of the wound over the femoral region on 12th day, using a non-absorbable suture (Nylon 1), interrupted technique. Antibiotics (Amoxicillin- clavulanic acid 625 mg twice daily and metronidazole 500 mg three times a day) were administered, combined with dressing all 2 days. Post-operative recovery was uneventful. The patient was discharged on post-operative day 18. The groin wound healed properly 7 days after to be discharged. The ileostomy reversal was performed after six weeks from the exploratory laparotomy, any complication occurred.

Our study was conducted in a teaching hospital and the surgery was performed by a senior consultant general surgeon with a resident in general surgery as an assistant.

## 3 Discussion

A Richter hernia occurs when the anti-mesenteric wall of the intestine protrudes, causing strangulation without obstruction [7]. While the Richter's hernia is named after German surgeon August Gottlieb Richter, who described this type of hernia in 1778, the earliest known case is believed to have been reported in 1598 by German surgeon Wilhelm Fabry [1,2]. In 1986, Horbach described a series of 146 strangulated hernias, of which 45 were Richter's hernias. He noted that Richter's hernias, as opposed to other strangulated hernias, were more commonly associated with bowel necrosis (31 out of 45 Richter's) [8]. Richter's hernias occur most commonly diagnosed in patients 60 to 80 years of age and comprise up to 10 % of all strangulated hernias [1]. The most common locations are femoral hernias in 72 to 88 %, followed by inguinal hernias 12 to 24 %, and abdominal wall incisional hernias 4 to 25 % [7]. Features of intestinal obstruction are typically absent, especially when less than two-thirds of the circumference of the bowel is involved, which leads to late diagnosis or bowel ischemia [9]. There are also other complications, which have been described in the literature, including spontaneous fistulae from the affected bowel and necrotic skin, which can lead to EC fistula. Richter hernia was also described as a complication of a colonoscopy by Fluri [10]. Another report described a case where a Richter's hernia was misidentified as a groin abscess and was only later discovered upon exploration after feculent material started to drain from the inguinal abscess, which is similar to the case described in this report [11].

Because patients infrequently have obstructive symptoms with Richter's hernia, they tend to progress to gangrene than is typically seen in other types of strangulated hernias. Repair is typically approached in the pre-peritoneal fashion, with a mandatory laparotomy and resection of bowel if gangrenous or perforated or EC Fistula associated [7,12]. In some cases, like our case, the presentation is a persistent bulging mass in the groin which leads to misdiagnosis of inguinal abscess.

An EC Fistula, forms when there is an abnormal communication between the gastrointestinal tract and the skin (or wound). The most common cause of EC Fistula is iatrogenic [9]. Only about a quarter of these cases may occur spontaneously and do so most commonly in the setting of inflammatory bowel disease like Crohn's disease [9,12]. Even in the most experienced hands and specialized centres, mortality remains high at 5–15 % [13]. Fistulae are associated with high morbidity and mortality. They equally attract high healthcare costs, pose a huge burden to patients psychologically, and offer a challenging problem to surgeons in terms of care and operative repair [14].

The diagnosis of Richter's hernia clinically is challenging. Many of the previous cases [15] were confirmed during surgery. Detailed clinical history, thorough physical examination and radiology may help in the early diagnosis of the patients. Richter's hernia poses diagnostic challenges mainly due to its silent presentation with absence of features of intestinal obstruction and this may only be made during surgery. The early and mild symptoms, such as abdominal pain and slight malaise, may not be conclusive, resulting in delayed diagnosis [2,4]. In the femoral and inguinal regions, the hernia may be misdiagnosed as an abscess as in our case report, inguinal lymphadenitis, or may present as necrotizing fasciitis, Fournier's gangrene [4]. The investigation of choice of an EC fistula is a Fistulogram with urograffin or a Magnetic resonance fistulogram, and both investigations give similar results as reported by Hajong et al. [4,15]. The mentioned investigations were not available within our hospital for this case and therefore abdominal ultrasound scan and x-ray were used.

In patients with Richter's hernia, urgent surgical exploration with bowel resection and primary anastomosis is usually mandated [3,10], in our case an ileostomy was made, explained by the peritonitis.

EC Fistula presents as a wound discharging enteric contents, with pain and abdominal tenderness, distension, or complications that might include sepsis, malnutrition, and electrolyte imbalances [14]. The basis of management focuses on the prevention and treatment of sepsis, control of fistula effluent in addition to fluid and nutritional support. Association of ECF and hernia, peritonitis and other complications indicate an early surgery to prevent severe complications [5]. The abscess aspect in our report is explained by: After strangulation, there is an exudation of a bloody fluid into the hernial sac, and with impairment of integrity of the bowel, infection of the fluid. As the sac wall and sub cutaneous tissues becomes infected and edematous, the skin will appear shiny, redness and swollen [2].

Notes:

✓ Development of an EC fistula in the setting of a Richter's hernia may allow decompression of any obstructed bowel. However, this poses a high risk of peritoneal contamination, peritonitis, sepsis, and overall increased morbidity and mortality risk.
✓ In this particular setting, urgent surgical exploration with bowel resection and anastomosis or ostomy creation is necessary to mitigate these risks.

## 4 Conclusion

A Richter hernia is a rare entity that has the potential for serious morbidity and mortality if not diagnosed in a timely fashion. Early detection and surgical treatment are paramount to improve outcomes. This case highlights how an incarcerated Richter's femoral hernia in a female misdiagnosed as an abscess delayed treatment, increased patient morbidity with development of an enterocutaneous fistula and peritonitis, and mandated surgical exploration to control sepsis and repair the hernia.

## Consent

Written informed consent was obtained from the patient for publication and any accompanying images. A copy of the written consent is available for review by the Editor-in-Chief of this journal on request.

## Ethical approval

It was not necessary because not applicable, there was any deviation from the standard ethical requests.

## Funding

There was no external funding sourced for this report.

## Guarantor

Baanitse Munihire Jeannot.

## Research registration number

1. Name of the registry: Not applicable
2. Unique identifying number or registration ID: Not applicable
3. Hyperlink to your specific registration: Not applicable.

## CRediT authorship contribution statement

BMJ, MAB, and MM managed the patient and wrote the first draft. RMW, JM and FKS helped in editing and reviewing the paper. All authors read and approved the final version to be published.

## Declaration of competing interest

The authors declare no conflict of interest.

## Data Availability

Not applicable.
